# The complete chloroplast genome of *Gerbera piloselloides* (L.) Cass., 1820 (Carduoideae, Asteraceae) and its phylogenetic analysis

**DOI:** 10.1515/biol-2025-1070

**Published:** 2025-03-25

**Authors:** Wentao Sheng

**Affiliations:** Department of Biological Technology, Nanchang Normal University, Nanchang, 330032, Jiangxi, China

**Keywords:** *Gerbera piloselloides*, chloroplast genome, SSR, codon, relationship

## Abstract

*Gerbera piloselloides* (L.) Cass., 1820 of the genus *Gerbera* is of importance in Chinese ethnic medicine. In this research, the whole genome DNA of *G. piloselloides* was extracted and sequenced using the Illumina NovaSeq platform, its chloroplast genome was assembled and annotated, and its sequence characteristics were analyzed using bioinformatic methods. The results showed that its chloroplast genome has a length of 151,871 bp and contains 133 annotated genes, consisting of 88 protein-coding genes, 8 rRNA genes, and 37 tRNA genes. In total, 202 simple sequence repeat sites and 43 long repeats were detected in *G. piloselloides*, mainly consisting of mono-nucleotide and tri-nucleotide repeats, with A/T as the major base composition. The chloroplast genome of *G. piloselloides* contains 22,772 codons, with leucine-coding codons being the most abundant. Comparative genomics showed that the genome structure, composition and variation were basically the same in the Asteraceae family. The phylogenetic tree analysis indicated a close relationship between the genus *Atractylodes* and *Gerbera,* consistent with the morphological classification. The research of the *G. piloselloides* chloroplast genome will lay a foundation for species discrimination, genetic evolution analysis, and DNA barcode construction in *Gerbera* plants.

## 1 Introduction

The chloroplast is a plant cell organelle enclosed by a double membrane that contains chlorophyll and can complete photosynthesis, which plays an important regulatory role in plant growth [1]. The chloroplast genome usually exists in the form of double stranded circular molecules, and most higher plants have a tetrad structure, which determines the conservatism of higher plants in gene expression, species formation, and other aspects [2,3]. The widespread application of high-throughput sequencing has resulted in an increasing number of plant chloroplast genome sequences [4]. To date, more than 55,351 plastid genome sequences have been uploaded to NCBI (www.ncbi.nlm.nih.gov/home, up to November 15, 2024). Compared to the nuclear genome, the chloroplast genome is more conserved in genome structure, length, GC content, gene sequence, and gene quantity [5,6]. Chloroplast genome studies demonstrated that the chloroplast genome has a wide range of applications in species identification, systematic evolution, and population genetics research. Sheng [7] carried out the molecular marker development and discriminated the plant of *Asparagus cochinchinensis* with chloroplast genome. He et al. [8] conducted research on the chloroplast genome sequencing analysis of *Cucurbita ficifolia* in the family Cucurbitaceae. Song et al. [9] provided the chloroplast genome sequence of eight *Delphinium* species, and performed a comparative analysis of this genus. Wang et al. [10] obtained molecular markers of pan-chloroplast genome sequences from the maize germplasm resources. He et al. [11] conducted research on the chloroplast genome of *Taraxacum* and developed molecular markers to distinguish their weedy relatives. Moreover, the chloroplast genome provides more molecular markers to accurately identify and distinguish different species and it is, therefore, known as the “super barcode” for species identification [12]. *Gerbera piloselloides* Cass. is a plant of the genus *Gerbera*, belonging to the Asteraceae family. There are approximately 80 species of this genus worldwide, mainly distributed in Africa, followed by East Asia [13]. There are approximately 20 species in China, mainly distributed in southwestern provinces, such as Guizhou, Yunnan, and Guangxi. *G. piloselloides* is a commonly used medicinal plant among ethnic minorities in China, promoting lung function, relieving sweating, cough, and promoting diuresis, Qi and blood circulation. But the variety of *Gerbera* plants used for medicinal purposes in various regions is not standardized [14]. This not only hinders the implementation of unified drug management nationwide but also poses great risks to drug safety. And research on *G. piloselloides* has mainly focused on its chemical composition [15,16,17]. Little research was concentrated on chloroplast genomes in plants of the genus *Gerbera*, and only 36 single nucleotide sequences have been obtained from NCBI (https://www.ncbi.nlm.nih.gov/nuccore/?term=Gerbera%20piloselloides). The only chloroplast genome of *Gerbera jamesonii* (NC:046760.1) was registered in this genus. In this research, the complete chloroplast genome sequence of *G. piloselloides* was successfully obtained. And my primary objectives are to (1) investigate the plastome structure and sequence divergence of *G. piloselloides*; (2) identify simple sequence repeats (SSRs) and divergence hotspots of *G. piloselloides*; and (3) preliminarily elucidate the phylogenetic relationships among the Chinese group of medicinal species and related taxa within Asteraceae.

## 2 Materials and methods

### 2.1 Sampling, DNA extraction, and sequencing

*G. piloselloidis* was sampled from the campus of Guizhou University in July, 2023 (26°25′39.62″N, 106°40′5.81″E). Fresh leaves (100 mg) were ground, and whole-genome DNA was extracted using the CTAB method [18]. The sampled DNA with a concentration greater than 200 ng/µL and an A260/A280 value between 1.8 and 2.0 was sent to Nanjing Genepioneer Biotechnologies Co., Ltd for quality inspection. Qualified DNA was used for library construction, and Illumina double-end sequencing was performed in Illumina NovaSeq platform of Genepioneer Biotechnologies Co. [19].

### 2.2 *De novo* assembly, gap filling, and gene annotation

The raw data were filtered and obtained with adapters for subsequent assembly. The chloroplast genome sequences were assembled using CLC Microbial Genomics Module 24.1.1 (https://digitalinsights.qiagen.com/clc-microbial-genomics-module-latest- improvements/) and Perl scripts to run NOVOPlasty v4.2 with default parameters [20]. BLAST (https://blast.Ncbi.nlm.nih.gov/) was performed to determine the sequence of contigs splicing (similarity index great than 90%), referring to NCBI’s published *G. jamesonii* (NC:046760.1). Then, the Geneious Prime 2024.0 software was used for splicing to obtain the final circular chloroplast genome and perform manual calibration (https://www. geneious.com/download-previous-versions). The resulting genome was annotated using online annotation software Annotation of Organellar Genomes [21] (https://chlorobox.mpimp-golm.mpg.de/geseq.html) and GetOrganelle [22] running Perl scripts (https://github.com/Kinggerm/GetOrganelle). The online servers version 2.0 (http://lowelab.ucsc.edu/tRNAscan-SE/) [23] were used to identify the tRNA gene, and the annotated sequence was uploaded to NCBI to register the sequence number. The OGDRAW version 1.3.1 visualization tool for chloroplast genome circle diagram was used to draw its genome map [24].

### 2.3 Repetitive sequence analysis

Repetitive sequences are important molecular sequences used for species identification, genetic diversity, population evolution, and other research [8]. The Tandem Repeats Finder v4.09 (https://tandem.bu.edu/trf/trf.html) was used to discriminate tandem repeat sequences [25]; the parameters were set as Match 2, Mismatch and Delta 7, PM 80, PI 10, Minscore 50, MaxPeriod 500. Then, the SSR Hunter v1.3 [26] was used to assess simple repeats of sequences with default parameters.

### 2.4 Analysis of codon preference

The protein-coding sequences were obtained from the chloroplast genes of the experimental materials using Geneious software (https://www.geneious.com/) and saved in FASTA format. The CodonW (https://codonw.sourceforge.net/) script was used to calculate the relative synonymous codon usage (RSCU) of protein-coding sequences. Codons with RSCU > 1 were considered preferred [27].

### 2.5 Chloroplast genome variation analysis

The *G. piloselloidis* chloroplast genome was compared and analyzed with published chloroplast genomes of the main Chinese representative medicinal plants from the Asteraceae family (*Atractylodes macrocephala*: NC_044671.1; *Chrysanthemum lavandulifolium*: NC_057202.1; *Artemisia annua*: NC_034683.1; *Taraxacum officinale*: NC_030772.1; *Arctium lappa*: NC_042724.1; *Xanthium Strumarium*: NC_042232.1; *Helianthus annuus*: NC_ 007977.1). The IRscope website (https://irscope.shinyapps.io/irapp/) was utilized to visualize the expansion and contraction of large single-copy (LSC), inverted repeat (IR), and small single-copy (SSC) regions in the chloroplast genomes of representative groups from the Asteraceae family.

Collinearity analysis of chloroplast genome was performed using the Geneious software (https://www.geneious.com/). Chloroplast genome alignment was conducted using the MAFFT version 7 software [28], and we manually proofread the alignment sequences using the BioLign 4.0.6 version software (https://www.softpedia.com/get/Science-CAD/BioLign.shtml), calculated chloroplast genome sequence nucleotide polymorphism (Pi) using the DnaSP version 6 software, set a search window length of 600 bp, step size of 200 bp, and plotted using the R program [29].

### 2.6 Phylogenetic analysis

To explore the phylogenetic relationship of the chloroplast genome of *G. Piloselloidis* and determine its taxonomic position, the online sequence alignment tool MAFFT v7 (https://mafft.cbrc.jp/) was used. The chloroplast genome sequence data of 31 Chinese medicinal plants were aligned and a sequence matrix was constructed using an alignment/server index. The principles for selecting these genera are common genera in the family, including the genus *Cirsium*, *Atractylodes*, *Chrysanthemum*, *Artemisia*, *Carthamus*, *Rhaponticum*, *Taraxacum*, *Inula*, *Arctium*, *Xanthium*, *Aster*, *Senecio*, *Lactuca*, *Dahlia*, *Eclipta*, *Gerbera*, *Bidens*, *Erigeron*, *Youngia*, *Ageratina*, *Mikania*, *Silybum*, *Saussurea*, *Ligularia*, *Stevia*, and *Helianthus*. The basis for species selection is the most common traditional Chinese medicine plant in the Asteraceae family in China, and *Platycodon grandiflorus* (Jacq.) A. DC. (KX352464.1) from the Campanulaceae family was also selected for the external group. Using maximum likelihood (ML) for phylogenetic analysis, a ML tree was generated with the integrated plugin IQ-TREE v2 [30]. Using IQ-TREE software’s ModelFinder to filter the most optimal model, set the self-expansion value to 1,000 to construct ML tree. And the ML tree was visualized and beautified using the Figtree v1.4.4 software (http://tree.bio.ed.ac.uk/software/figtree/).

## 3 Results

### 3.1 Basic characteristics

**Figure 1:**
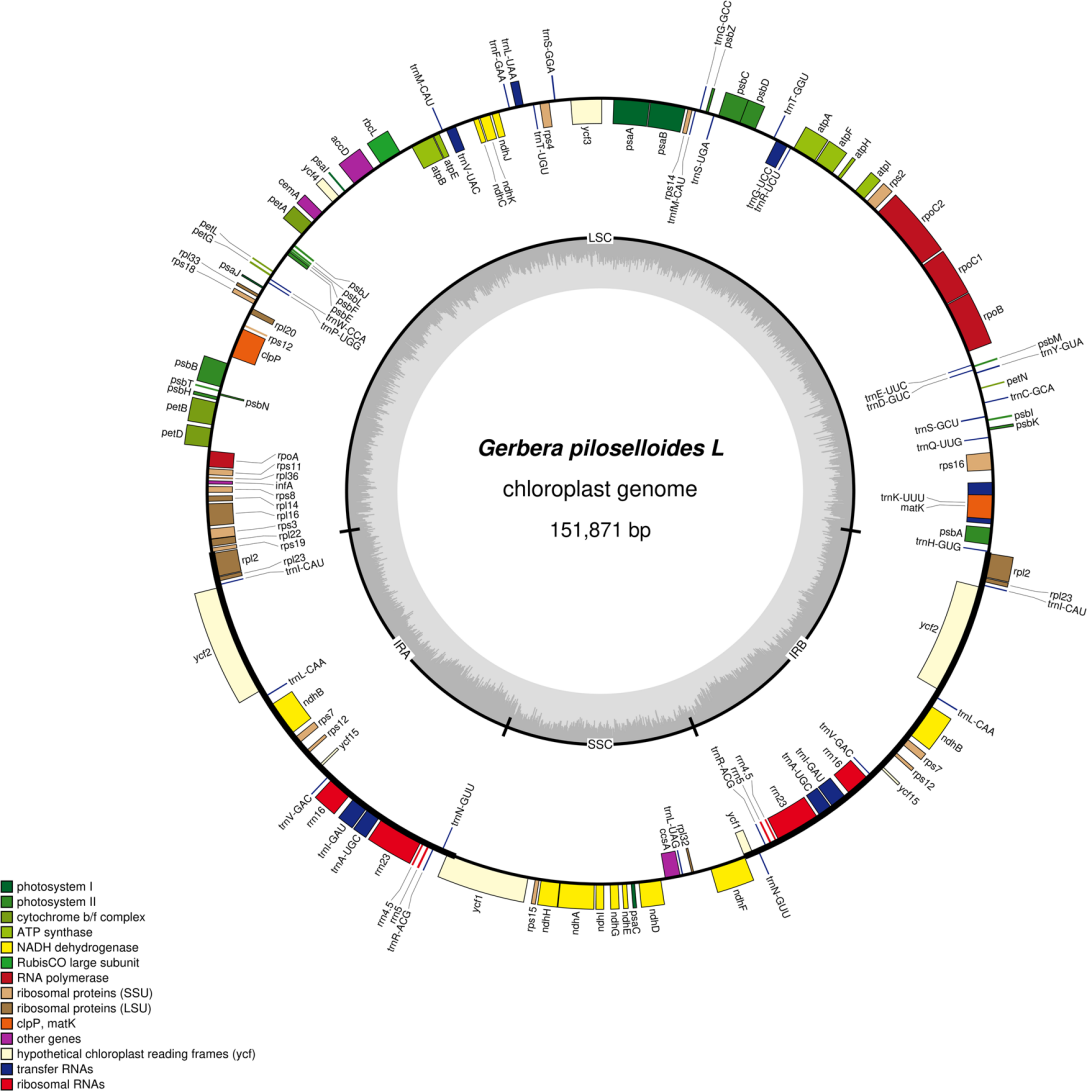
Chloroplast genome map of *Gerbera piloselloides*.

As shown in Table 1, the complete length of the chloroplast genome sequence of *G. piloselloidis* was 151,871 bp (GenBank accession number: PP473789), and has a typical four-segment structure. The lengths of the LSC region, the two IR regions, and SSC region were 83,468, 50,172, and 18,231 bp, respectively, and the GC content was 37.75%. There were 133 annotated genes in the chloroplast genome of *G. piloselloidis*, with 84 (63.15%), 36 (27.06%), and 13 genes (9.77%) in the LSC, IR, and SSC regions, respectively. There were 88 protein-coding genes, accounting for 66.16% of the total gene number; the number of ribosomal RNAs (rRNAs) was 8, accounting for 6.02%; and the number of transport RNA (tRNAs) was 37, accounting for 27.82%. As shown in Figure 1, four rRNA genes (4.5S, 5S, 16S, and 23S), seven tRNA genes (*trnV-GAC*, *trnI-GAU, trnI-CAU*, *trnL-CAA*, *trnA-UGC*, *trnN-GUU*, and *trnR-ACG*), and seven protein-coding genes (*rpl2*, *rpl23*, *rps12*, *ycf2*, *ndhB*, *ycf15*, and *rps7*) are located in the IR region. All 18 genes had two copies in the chloroplast genome.

**Table 1:** Basic characteristics of chloroplast genome in *Gerbera piloselloides*

Feature	Numerical value
GC content (%)	37.75
LSC length (bp)	83,468
SSC length (bp)	18,231
IR length (bp)	25,086
Gene number	133
Gene number in LSC regions	84
Gene number in SSC regions	13
Gene number in IR regions	36
Protein-coding gene number	88
Protein-coding gene percentage (%)	61.16
rRNA gene number	8
rRNA percentage (%)	6.02
tRNA gene number	37
tRNA percentage (%)	27.82
Length (bp)	151,871

Note: LSC: large single-copy; IR: inverted repeat; SSC: small single-copy; bp: base pair.

Based on the classification of all gene functions, most genes associated with photosynthesis and self-replication were found in the chloroplast genes of *G. piloselloides* (Table S1). Photosynthesis-related genes involves six major subunits: NADH dehydrogenase, ATP synthase, cytochrome b/f complex, photosystem I, photosystem II, and ribose diphosphate oxygenase/carboxylase genes. And the number of genes related to photosystem II subunits was the highest, with a total of 15 genes. But only one gene (*rbcL*) was associated with the ribose diphosphate oxygenase/carboxylase subunit. There are five categories of self-replication-related genes: large subunits of ribosomes, DNA-dependent RNA polymerase, small subunits of ribosomes, rRNA genes, and tRNA genes. The tRNA gene family contained the highest number of genes (30), whereas the DNA-dependent RNA polymerase class had the lowest number of genes, with only four (*rpoA, rpoB, rpoC1,* and *rpoC2*). Further analysis of the chloroplast genes of *G. piloselloidis* showed that most genes did not have introns (Table S2). In the present study, only 23 genes in the chloroplast genome of *G. piloselloidis* had introns. Except for two introns in *ycf3* and *clpP*, all other genes contained one intron. The intron of *trnK-UUU* was the longest, reaching 2,526 bp, and the intron of *trnL-UAA* was the smallest (431 bp).

### 3.2 Condon usage bias

**Figure 2:**
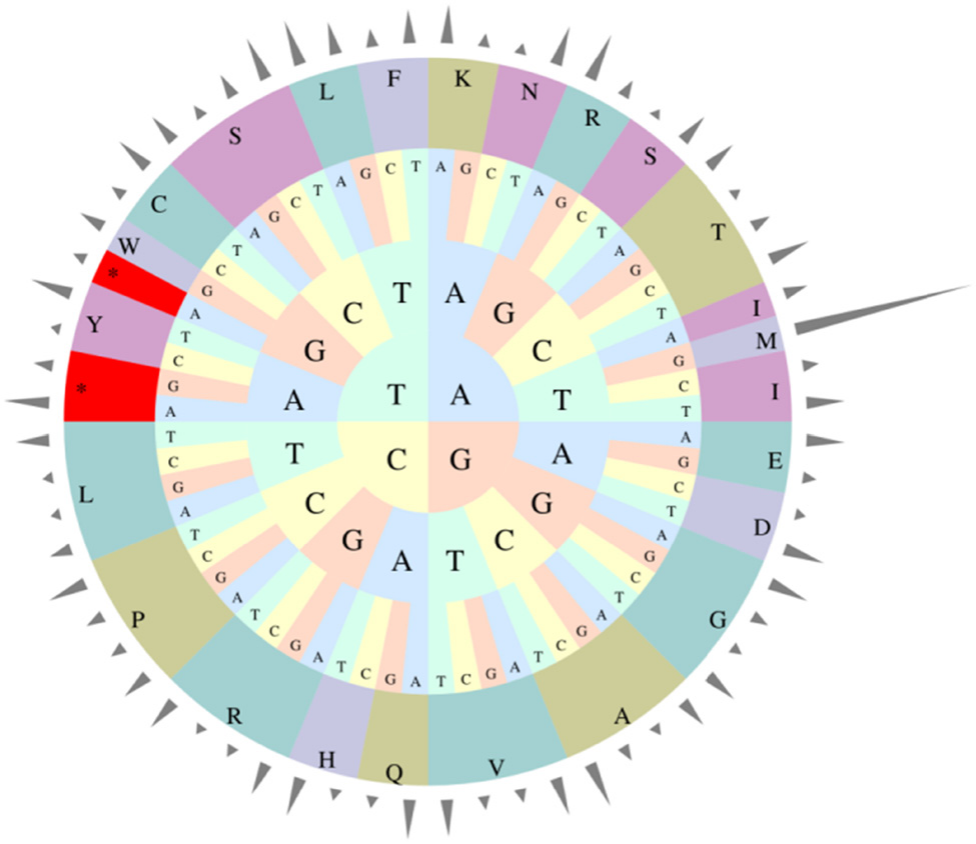
Codon usage in chloroplast genome of *Gerbera piloselloidis*.

In the *G. piloselloidis* chloroplast genome, 61.16% of the sequences were made up of gene-coding regions. Statistical analysis of the codons from all protein-coding genes showed that 2,416 (10.61%) codons encoded leucine (Leu), which has the highest coding rate, and only 251 (1.1%) codons encoded cysteine (Cys), which has the lowest coding rate. In the protein-coding region, the AT contents in the first, second, and third codons were 55.47, 62.88, and 71.63%, respectively (Figure 2). This codon-encoding preference, with high AT content in the third position, is extremely common in the chloroplast genomes of other higher plants [5].

### 3.3 Repetitive sequence analysis

**Figure 3:**
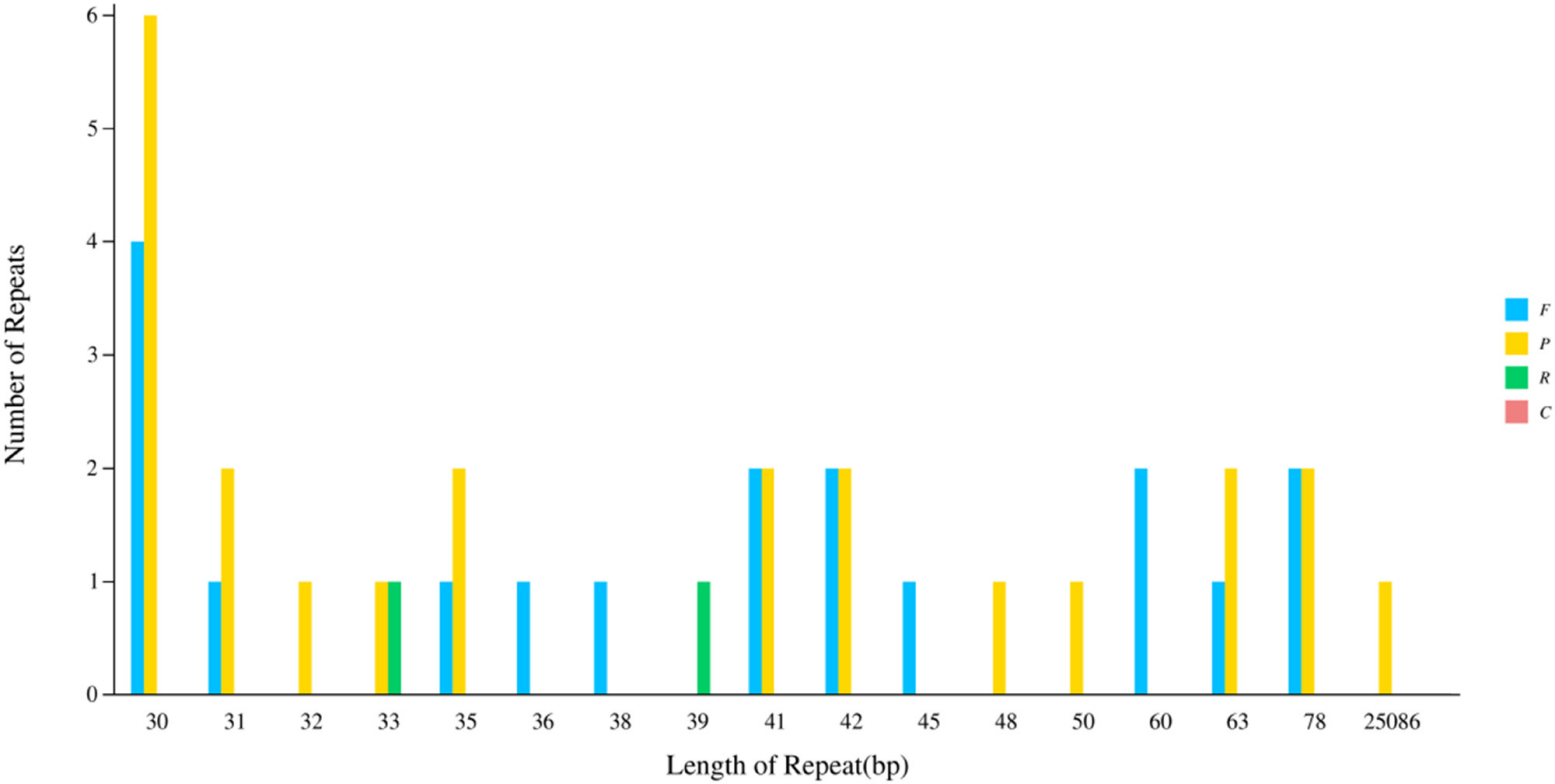
The long fragment repeats of *Gerbera piloselloidis.* Note: F: forward repeats; P: palindromic repeats; R: reverse repeats; and C: complementary repeats.

**Figure 4:**
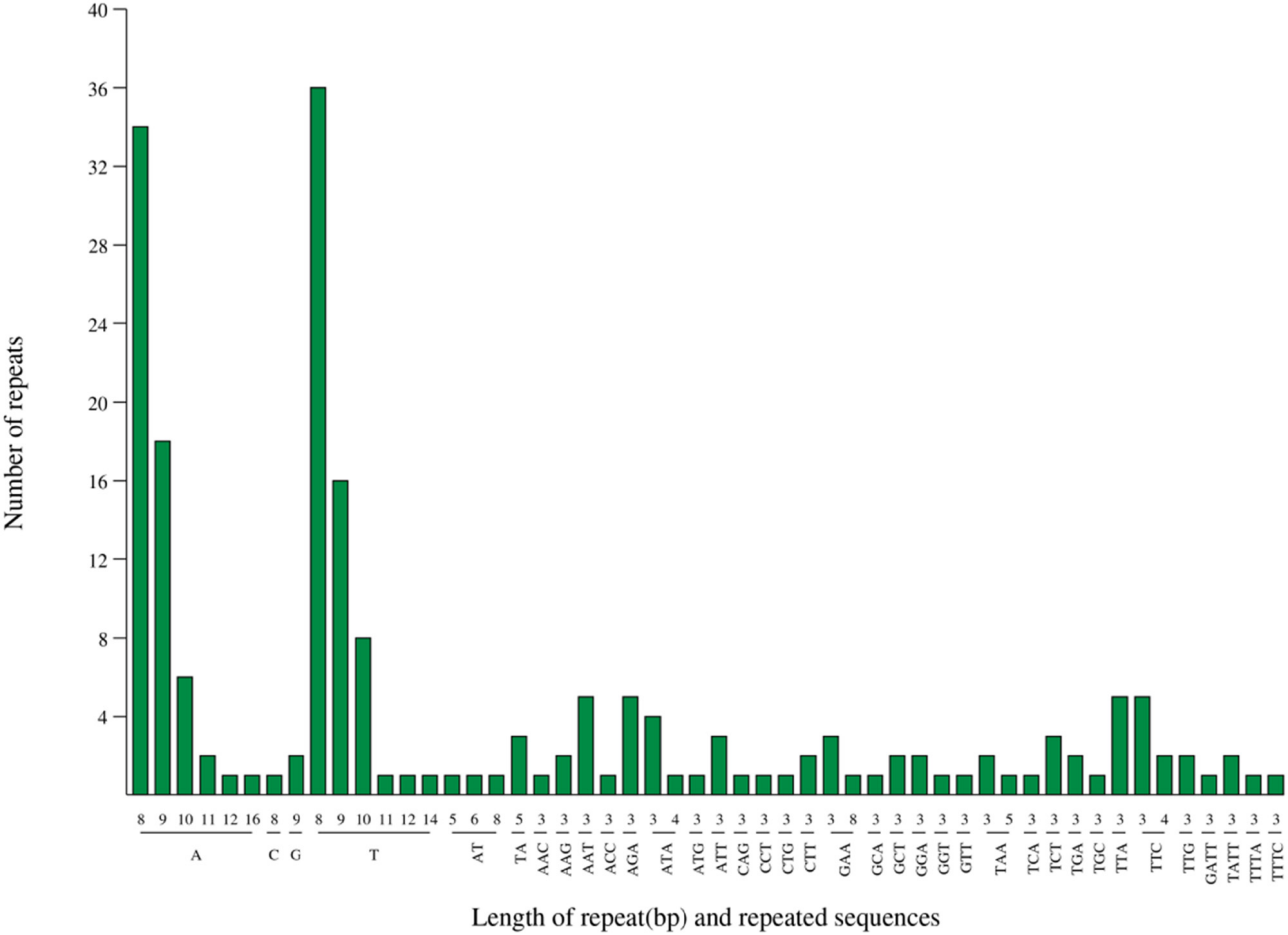
The SSR type and length of *Gerbera piloselloidis.*

In total, 43 tandem repeats (T), including 18 forward repeats (F), 23 palindromic repeats (P), and two reverse repeats (R), were identified through repeat sequence analysis of *G. piloselloidis*, but no complementary repeats (C) were discovered (Figure 3, Table S3). In total, 202 information loci were excavated in the chloroplast genome of *G. Piloselloidis*, containing 128 single nucleotides, six dinucleotides, 63 trinucleotides, and 5 tetranucleotides (Figure 4). Only 15 of them were in the coding area.

### 3.4 Chloroplast genome comparative analysis and boundary position difference

To compare the differences in chloroplast genomes between *G. piloselloidis* and its closely related species, we compared the basic information on chloroplast genomes between *G. piloselloidis* and a representative group of Chinese medicinal plants in Asteraceae (Table 2). The chloroplast genomes of *G. Piloselloidis* and its seven closely related species were highly similar, with a total length ranging from 150,952 bp (*Artemisia annua*) to 153,256 bp (*Atractylodes macrocephala*), all of which are typical tetrad structures, and the GC content was also very close (37.5–37.7%). The gene compositions and quantities in these species were similar.

**Table 2:** Comparison of eight representative chloroplast genomes in Asteraceae medicinal species

Genome structure	*Gerbera piloselloides* PP473789	*Atractylodes macrocephala* NC_044671.1	*Chrysanthemum lavandulifolium* NC_057202.1	*Artemisia annua* NC_034683.1	*Taraxacum officinale* NC_030772.1	*Arctium lappa* NC_042724.1	*Xanthium strumarium* NC_042232.1	*Helianthus annuus* NC_007977.1
Genome size/bp	151,871	153,256	151,085	150,952	151,324	152,708	151,897	151,104
LSC length/bp	83,468	84,290	83,034	82,772	83,895	83,764	83,846	83,530
SSC length/bp	18,231	18,674	18,279	18,268	18,567	18,582	17,900	18,308
IR length/bp	25,086	25,146	24,886	24,956	24,431	25,181	24,905	24,633
GC content (%)	37.7	37.7	37.5	37.5	37.7	37.7	37.5	37.6
Number of genes	133	125	131/132	133	133	132	132/133	136/137
Protein-coding gene	88	88	88 + 1pseudogene	88	88	87	87 + 1pseudogene	85 + 1pseudogene
rRNA	8	8	8	8	8	8	8	8
tRNA	37	29	35	37	37	37	37	43

**Figure 5:**
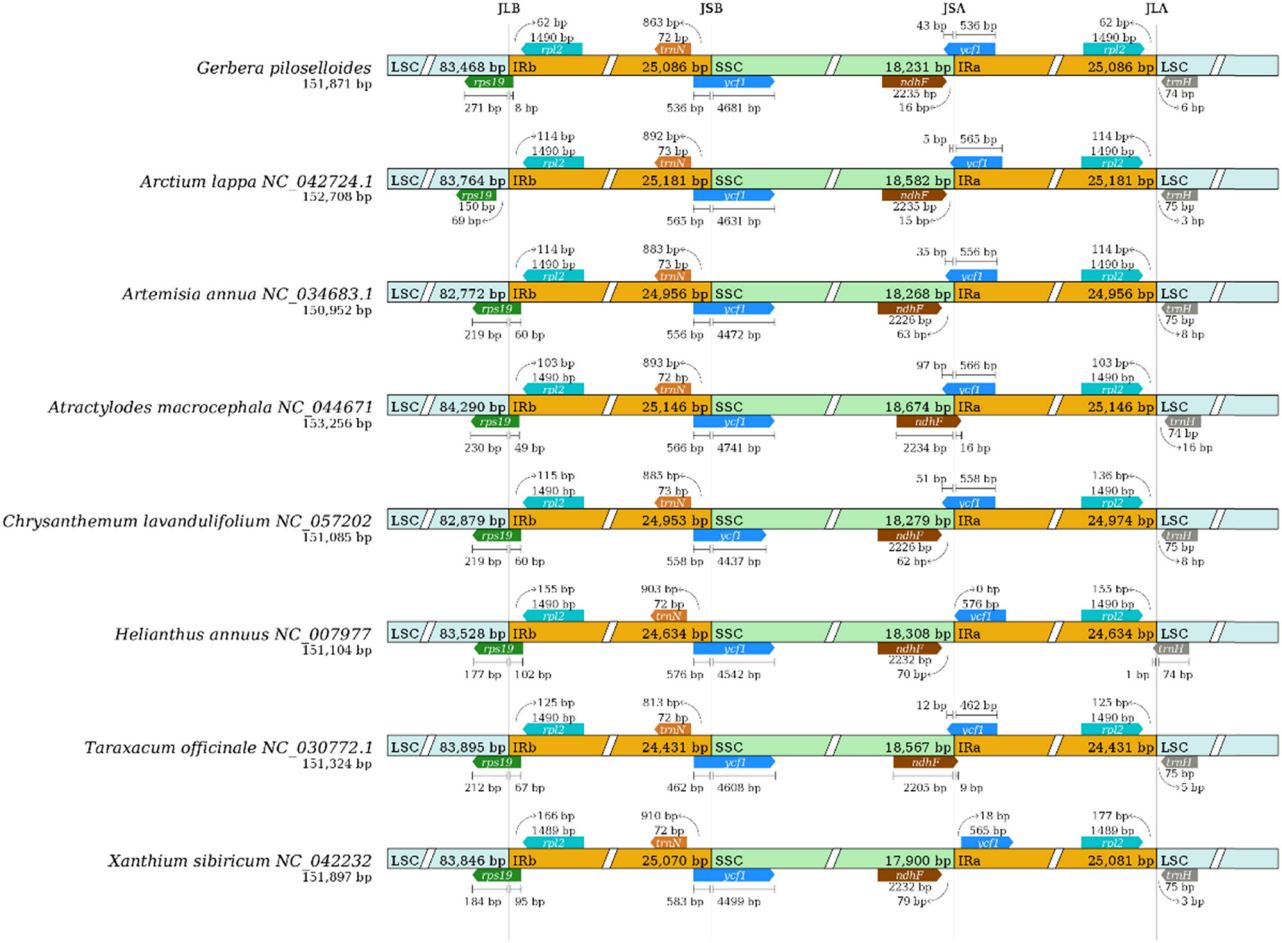
Boundary comparison of chloroplast genome in the Asteraceae.

The boundary positions of the eight Asteraceae species were compared and analyzed using IRscope (https://irscope.shinyapps.io/IRplus/). Among the four regions of the chloroplast genomes, the IR region was relatively conserved, with sequence sizes ranging from 24,431 to 25,181 bp. As shown in Figure 5, the IR boundary of *G. Piloselloidis* chloroplast genome is similar to those of the other seven species. The LSC/IRb, IRb/SSC, and SSC/IRa boundaries were located within the *rps19*, *ycf1,* and *trnH* gene, respectively. At the LSC/IRb boundary, *rps19* for *Arctium lappa* which was 69 bp away from this locus, and this gene in other seven Asteraceae species spanned this locus. At the IRb/SSC and SSC/IRa loci, y*cf1* spanned these two loci, with 462–583 bp. At the IRa/LSC locus, *trnH* was located far away from the locus from *Xanthium strumarium* to *Atractylodes macrocephala*, with sizes ranging from 1 to 16 bp. However, in *Helianthus annuus*, *trnH* spanned the locus by 1 bp.

### 3.5 Genomic structure and molecular sequence variations

**Figure 6:**
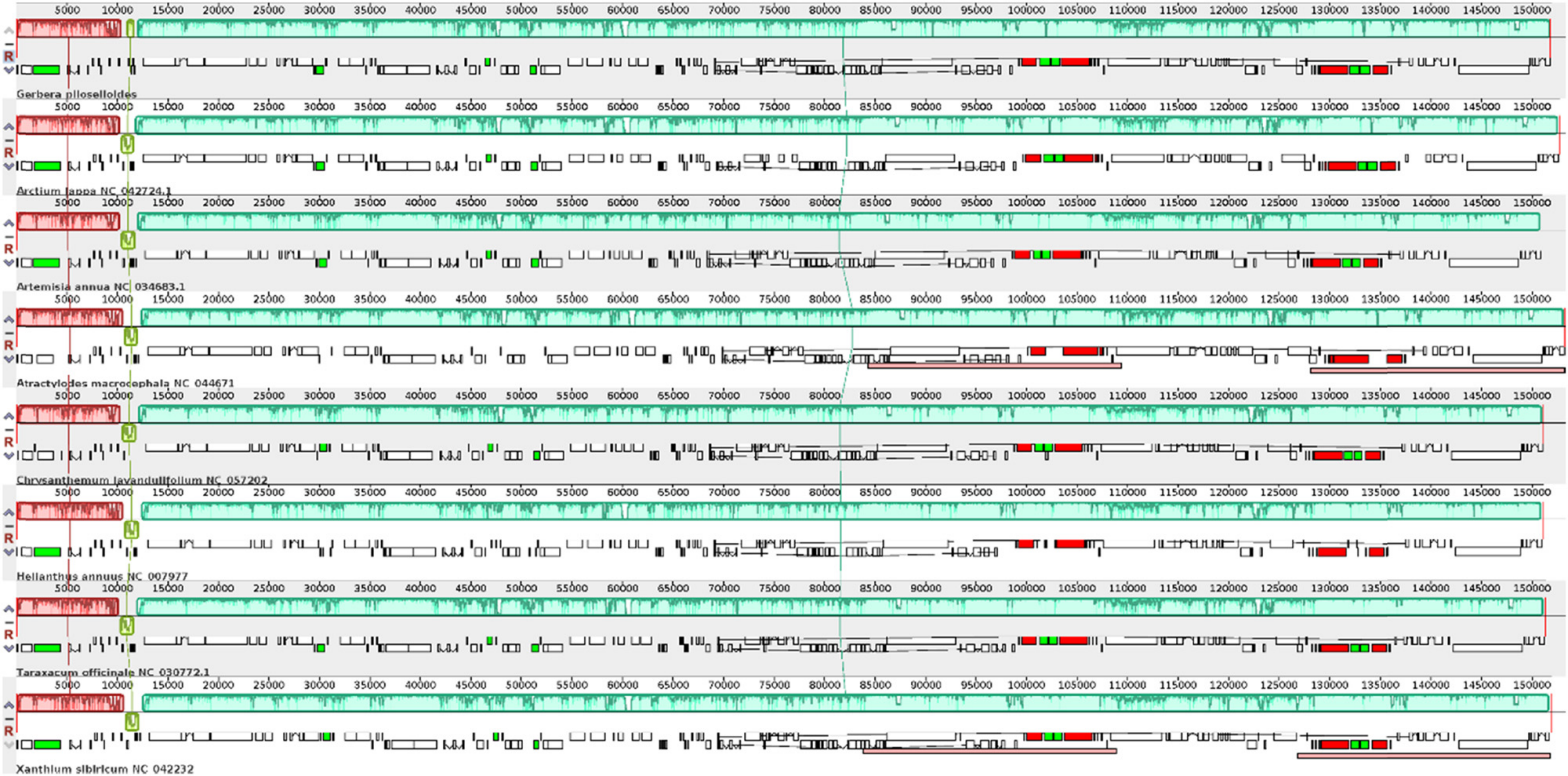
Alignment of eight representative chloroplast genomes structure of Asteraceae species.

**Figure 7:**
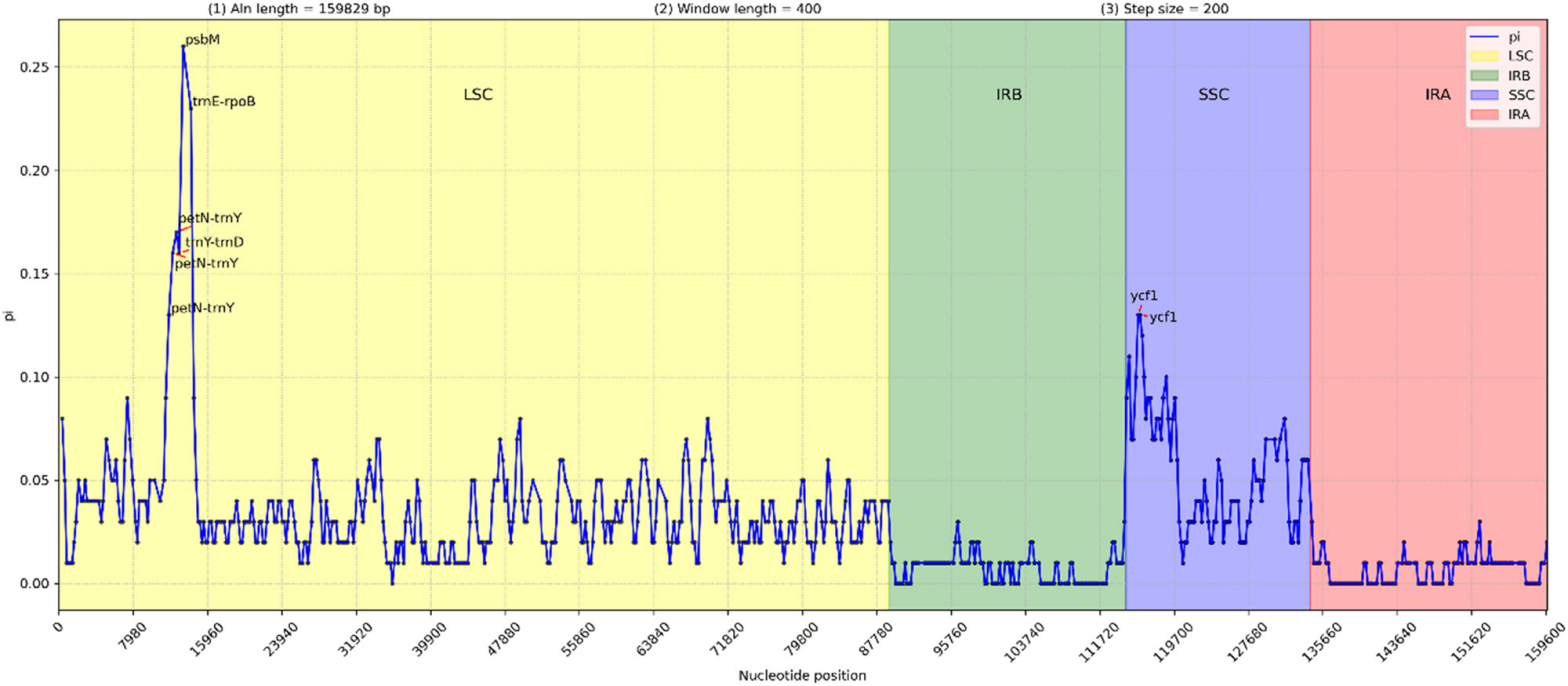
The *P*_i_ value of eight representative chloroplast genome sequences of Asteraceae species.

The rearrangement and collinearity of the Asteraceae chloroplast genomes were analyzed using the Geneious software (Figure 6). And the chloroplast genome structures of the eight Asteraceae species were highly similar, with consistent numbers and arrangements of all genes, and no gene rearrangement or inversion events were found. Nucleotide polymorphisms in the chloroplast genome sequence of Asteraceae species were analyzed using the DnaSP software (Figure 7). In total, 10,879 polymorphic sites were determined in the eight genomes, accounting for 7.16% of the total sequence length. Nucleotide polymorphism values (*P*_i_) ranged from 0 to 0.25536, with an average value of 0.02738. The average *P*_i_ in the LSC, SSC, and IR regions were 0.01736, 0.0293, and 0.00463, respectively. Seven highly variable regions (*P*_i_ > 0.1) were detected, most of which were intergenic region. And four sequence fragments (*petN-trnY*, *trnY-trnD*, *trnE-rpoB,* and *psbM*) were located in the LSC region, and one sequence (*ycf1*) was located in the SSC region. The highest polymorphic site *P*_i_ value (*psbM*) in the LSC region was 0.25536, indicating higher nucleotide polymorphisms in the SSC and LSC than in the IR region.

### 3.6 Phylogenetic analysis

**Figure 8:**
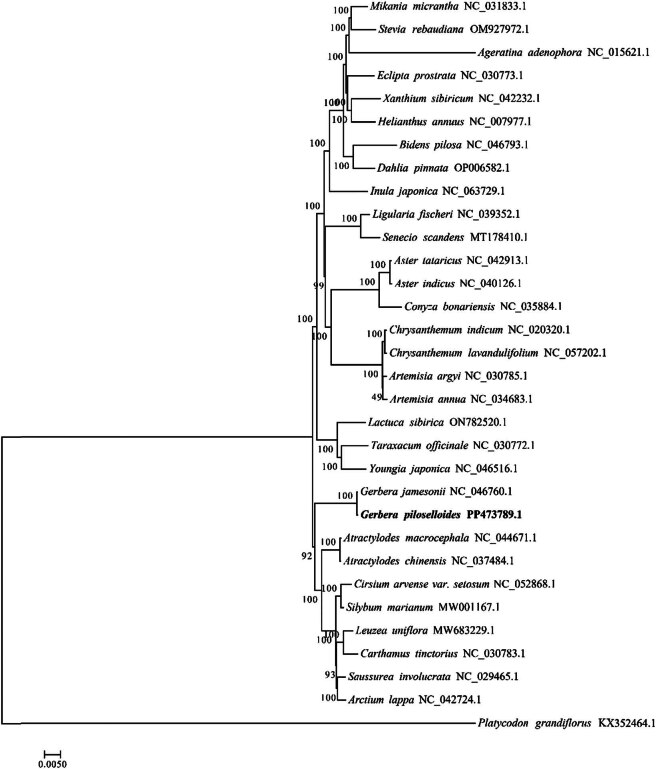
Phylogenetic trees based on the whole-genome sequences of chloroplast genomes of 31 Asteraceae medicinal species.

To distinguish the phylogenetic position of *G. piloselloidis*, a reference was made to the chloroplast sequences of 31 other Chinese herbal medicinal plants in Asteraceae already published by NCBI, and the whole genome sequences were selected for phylogenetic analysis. The results were as follows: the support rate for clustering was high, with a test score of 100% for 24 nodes, indicating a high reliability of the clustering results. As shown in Figure 8, the 32 plants can be divided into two main categories. The first category includes 13 plant species, containing the genera *Lactuca*, *Taraxacum*, *Youngia*, *Gerbera*, *Atractylodes*, *Cirsium*, *Silybum*, *Rhaponticum*, *Carthamus*, *Saussurea,* and *Arctium*. The second category includes 18 plant species from the genera *Mikania*, *Stevia*, *Ageratina*, *Eclipta*, *Xanthium, Helianthus*, *Bidens*, *Dahlia*, *Inula*, *Ligularia*, *Senecio*, *Aster*, *Erigeron*, *Chrysanthemum,* and *Artemisia*. According to the cluster diagram, the closest phylogenetic relationship was observed between *G. piloselloidis* and *Gerbera jamesonii* of the same genus, *Gerbera*, followed by *Atractylodes macrocephala* and *Atractylodes chinensis* of the genus *Atractylodes*. *Gerbera* and *Atractylodes* belong to the Carduoideae subfamily, and the above results are consistent with traditional taxonomy.

## 4 Discussion

Chloroplasts are semi-autonomous organelles within plant cells that are the main sites of photosynthesis, and this organelle plays an important role in plant growth. Sequencing of the chloroplast genome provides a basis for further understanding chloroplast gene expression, photosynthesis, cytoplasmic interactions, and other related research [1,31]. Herein, we reported the assembly and annotation of the high-quality *G. Piloselloidis* chloroplast genome. The chloroplast genome size of *G. piloselloidis* was 151,871 bp, and the length of the IR region was 25,086 bp. Within the range of 120–180 kb and 20–30 kb in angiosperms [4,32], it had a typical circular tetrad structure of the angiosperm chloroplast genome. The GC content was 37.7%, and it comprised 133 genes. Among these genes, 21 genes (*trnK-UUU, rps16, rpoC1, atpF, trnG-UCC, trnL-UAA, petB, petD, trnV-UAC, rps12, rpl16, rpl2, ndhB, trnA-UGC, ndhA, rps12, trnI-GAU, trnI-GAU, trnA-UGC, ndhB,* and *rpl2*) contained a single intron, and two genes (*clpP* and *ycf3*) contained two introns.

In total, 202 SSR loci were determined, including 36 repeating units. Among them, the highest number of mononucleotide repeats were found, with a total of 128, accounting for 63.37% of the total SSR number; trinucleotide repeats, totaling 63, accounting for 31.18% of all SSRs; and once again, there were 6 dinucleotide repeats and 4 tetranucleotide repeats, without pentanucleotide and hexanucleotide repeats. A large number of SSRs with dinucleotide motifs are also relatively rare in other species [33], which may be due to different screening criteria. AG, AT, AAG, AGG, and AGC were the main types of repetitive motifs, which is consistent with previous reports [34]. The A/T and AT/AT repeat units accounted for 66.9% of all SSR sites, and polyA or polyT is an important reason for the high AT content in chloroplast genomes. Most perfect SSR sequences are based on short sequences of 6–8 bp in length, which are related to a large number of trinucleotide-based SSRs [35].

This highly variable region of the chloroplast genome sequence could serve as a molecular marker for plant species identification [36]. Size differences and nucleotide variations in the single-copy regions between the chloroplast genomes of *Gerbera* species were greater than those in the IR region. Among the five nucleotide hypervariable regions with *P*_i_ > 0.1 identified in this study, three (*petN-trnY, trnY-trnD,* and *trnE-rpoB*) were located in the intergenic region, and two were located on *psbM* and *ycf1* gene, which is consistent with the results for the genus *Artemisia* [37]. The chloroplast *ycf1* is crucial for plant environmental adaptability, and nucleotide sequence polymorphisms reflect the adaptation of plants to different environments [4]. These polymorphic regions can be made to develop powerful candidate sites for taxonomic research and genetic diversity analysis of *Gerbera* species.

Codon preference refers to the uneven use of synonymous codons and it has an important role in the evolution of biological genomes. This is usually reflected in the frequency of the RSCU values [38]. RSCU analysis showed that the codons with the highest and lowest RSCU values were AGA (2.10) and CGA (0.47), respectively, both of which encode Arginine. Among the codons, 31 had RSCU values greater than 1, of which 27 ended in A/U, indicating that the chloroplast genome of *G. piloselloidis* prefers codons ending in A/U. The codons with A/T as the first base accounted for 55.47% of the total codon count, the second for 62.88%, and the third for 71.63% in *G. piloselloidis*. Furthermore, this codon-encoding preference with high AT content in the third position is extremely common in the chloroplast genomes of other higher plants [39].

*G. piloselloidis* is often used as an ornamental plant abroad; however, little research has been conducted on its use. However, it is widely used in ethnic medicine in Southwestern China. Studying the chemical composition and pharmacological activity of *G. piloselloidis* can promote the standardization of related medicinal materials, provide theoretical guidance, and facilitate further research and development of this ethnic medicine [40]. And special SSR motifs and divergent hotspot regions identified from *G. piloselloidis* chloroplast provided reference for subsequent identification investigations. In order to more accurately identify *G. piloselloidis* in the Asteraceae family, it is necessary to conduct further research on these systematic relationships. Each variety is strictly distinguished based on its molecular sequence, and its identification is crucial to achieve the goal of authentic and safe drug use. A systematic evolutionary analysis of the complete genome sequence in the chloroplasts of 31 other published medicinal plants showed that *G. piloselloidis* has the closest phylogenetic relationship with *Gerbera jamesonii*, followed by *Atractylodes macrocephala* and *Atractylodes chinensis* of the genus *Atractylodes,* and the evolutionary analysis results are consistent with traditional taxonomy (https://www.iplant.cn/info/Asteraceae). The plastome-based phylogeny provided preliminary insights into the relationships among the Chinese group of medicinal species and related taxa within Asteraceae.

## 5 Conclusion

In conclusion, the *G. piloselloidis* chloroplast genome was sequenced and assembled in this research. The chloroplast genome structure, repeat sequences, codon preferences, and phylogenetic relationships of *G. piloselloidis* might provide a theoretical basis for phylogenetic analysis of plants in the genus *Gerbera*, and serve as important references for taxonomic research and phylogenetic analysis within the Asteraceae family. Overall, this study offers a wealth of informative genetic resources pertinent to *G. piloselloidis*, thereby enhancing our understanding of its evolution and laying a foundation for species identification, assessment of genetic population diversity, as well as the exploration and conservation of germplasm resources in Asteraceae.

## Supplementary Material

Supplementary Table
