# Supplementary material for: The complete chloroplast genome of Gerbera piloselloides (L.) Cass., 1820 (Carduoideae, Asteraceae) and its phylogenetic analysis
Source: Open Life Sci. 2025 Mar 25;20(1):20251070. doi: 10.1515/biol-2025-1070 (PMC11947664; doi:10.1515/biol-2025-1070)
Supplement: Supplementary Table [file biol-2025-1070-sm.pdf]

Supplementary material

Table S1: Gene list present in *G. Piloselloidis* chloroplast genome

Function	Family name	Gene
Genes for photosynthesis	Subunits of photosystem I	*psaA,psaB,psaC,psaI,psaJ*
	Subunits of photosystem II	*psbA,psbB,psbC,psbD,psbE,psbF,psbH,psbI,psbJ,psbK,psbL,psbM,psbN,psbT,psbZ*
	Subunits of NADH dehydrogenase	*ndhA*,ndhB*(2),ndhC,ndhD,ndhE,ndhF,ndhG,ndhH,ndhI,ndhJ,ndhK*
	Subunits of cytochrome b/f complex	*petA,petB*,petD*,petG,petL,petN*
	Subunits of ATP synthase	*atpA,atpB,atpE,atpF*,atpH,atpI*
	Large subunit of rubisco	*rbcL*
	Subunits photochlorophyllide reductase	—
Self replication	Large subunit of ribosome	*rpl14,rpl16*,rpl2*(2),rpl20,rpl22,rpl23(2),rpl32,rpl33,rpl36*
	DNA dependent RNA polymerase	*rps11,rps12*(2),rps14,rps15,rps16*,rps18,rps19,rps2,rps3,rps4,rps7(2),rps8*
	Small subunit of ribosome	*rpoA,rpoB,rpoC1*,rpoC2*
	rRNA genes	*rrn16(2),rrn23(2),rrn4.5(2),rrn5(2)*
	tRNA genes	*trnA-UGC*(2),trnC-GCA,trnD-GUC,trnE-UUC,trnF-GAA,trnG-GCC,trnG-UCC*,trnH-GUG,trnI-CAU(2),trnI-GAU*(2),trnK-UUU*,trnL-CAA(2),trnL-UAA*,trnL-UAG,trnM-CAU,trnN-GUU(2),trnP-UGG,trnQ-UUG,trnR-ACG(2),trnR-UCU,trnS-GCU,trnS-GGA,trnS-UGA,trnT-GGU,trnT-UGU,trnV-GAC(2),trnV-UAC*,trnW-CCA,trnY-GUA,trnfM-CAU*
Other genes	Maturase	*matK*
	Protease	*clpP***
	Envelope membrane protein	*cemA*
	Acetyl-CoA carboxylase	*accD*
	c-type cytochrome synthesis gene	*ccsA*
	Translation initiation factor	*infA*
	other	—
Genes of unknown function	Conserved hypothetical chloroplast ORF	*ycf1(2), ycf15(2), ycf2(2), ycf3**, ycf4*

Notes: Gene*: Gene with one intron; Gene**: Gene with two introns; #Gene: Pseudo gene; Gene (2): Number of copies of multi-copy genes.

Table S2: The length of introns and exons in *G. Piloselloidis*

Gene	Location	Exon I(bp)	Intron I (bp)	Exon II (bp)	Intron II (bp)	Exon III (bp)
trnK-UUU	LSC	37	2,526	36		
rps16	LSC	40	860	215		
rpoC1	LSC	432	744	1,638		
atpF	LSC	145	706	410		
trnG-UCC	LSC	23	706	48		
ycf3	LSC	124	742	230	704	153
trnL-UAA	LSC	37	431	50		
trnV-UAC	LSC	38	571	37		
rps12	IRa	114	—	243		
clpP	LSC	71	635	291	657	229
petB	LSC	6	771	642		
petD	LSC	8	706	475		
rpl16	LSC	9	992	399		
rpl2	IRb	391	665	434		
ndhB	IRb	777	670	756		
rps12	IRb	243	—	114		
trnI-GAU	IRb	43	943	35		
trnA-UGC	IRb	38	821	35		
ndhA	SSC	553	1,029	539		
trnA-UGC	IRa	38	821	35		
trnI-GAU	IRa	43	943	35		
ndhB	IRa	777	670	756		
rpl2	IRa	391	665	434		

Note: IR(inverted regions); LSC(large single copy); SSC(small single copy).

Table S3: The specific information of dispersed repeat distribution

ID	Repeat I Start	Repeat II Start	Type	Size (bp)	Distance	*E*-Value	Gene	Region
1	83469	126786	P	25086	0	0.00E+00	—	ir
2	90659	90677	F	78	-2	1.92E-33	*ycf2;ycf2*	IRb;IRb
3	90659	144586	P	78	-2	1.92E-33	*ycf2;ycf2*	IRb;IRa
4	90677	144604	P	78	-2	1.92E-33	*ycf2;ycf2*	IRb;IRa
5	144586	144604	F	78	-2	1.92E-33	*ycf2;ycf2*	IRa;IRa
6	90674	90692	F	63	0	7.63E-29	*ycf2;ycf2*	IRb;IRb
7	90674	144586	P	63	0	7.63E-29	*ycf2;ycf2*	IRb;IRa
8	90692	144604	P	63	0	7.63E-29	*ycf2;ycf2*	IRb;IRa
9	90659	90695	F	60	-2	7.77E-23	*ycf2;ycf2*	IRb;IRb
10	144586	144622	F	60	-2	7.77E-23	*ycf2;ycf2*	IRa;IRa
11	10562	11787	P	50	-2	5.64E-17	IGS	LSC;LSC
12	73565	73565	P	48	0	8.19E-20	*psbN;psbN*	LSC;LSC
13	90674	90710	F	45	0	5.24E-18	*ycf2;ycf2*	IRb;IRb
14	42780	116701	P	42	-1	4.23E-14	*ycf3;ndhA*	LSC;SSC
15	11730	11730	P	42	-2	2.60E-12	IGS	LSC;LSC
16	90659	90713	F	42	-2	2.60E-12	*ycf2;ycf2*	IRb;IRb
17	144586	144640	F	42	-2	2.60E-12	*ycf2;ycf2*	IRa;IRa
18	42781	97456	F	41	0	1.34E-15	*ycf3;*IGS	LSC;IRb
19	42781	137844	P	41	0	1.34E-15	*ycf3;*IGS	LSC;IRa
20	97456	116701	P	41	-1	1.65E-13	IGS;*ndhA*	IRb;SSC
21	116701	137844	F	41	-1	1.65E-13	*ndhA;*IGS	SSC;IRa
22	31235	31238	R	39	-3	5.30E-09	IGS	LSC;LSC
23	10462	10554	F	38	-3	1.96E-08	IGS	LSC;LSC
24	111177	111201	F	36	-3	2.65E-07	*ycf1;ycf1*	SSC;SSC
25	10470	11802	P	35	-3	9.71E-07	IGS	LSC;LSC
26	94413	116702	P	35	-3	9.71E-07	*ndhB;ndhA*	IRb;SSC
27	116702	140893	F	35	-3	9.71E-07	*ndhA;ndhB*	SSC;IRa
28	110864	113269	P	33	-3	1.30E-05	*ycf1;*IGS	SSC;SSC
29	31249	31230	R	33	-3	1.30E-05	IGS	LSC;LSC
30	10630	11900	P	32	-3	4.71E-05	IGS	LSC;LSC
31	10581	11787	P	31	-1	1.31E-07	IGS	LSC;LSC
32	7221	7248	F	31	-3	1.71E-04	IGS	LSC;LSC
33	10484	11792	P	31	-3	1.71E-04	IGS	LSC;LSC
34	8343	44540	P	30	0	5.63E-09	*trnS-GCU;trnS-GGA*	LSC;LSC
35	90671	90725	F	30	-1	5.06E-07	*ycf2;ycf2*	IRb;IRb
36	90671	144586	P	30	-1	5.06E-07	*ycf2;ycf2*	IRb;IRa
37	90725	144640	P	30	-1	5.06E-07	*ycf2;ycf2*	IRb;IRa
38	106518	106550	F	30	-2	2.20E-05	IGS	IRb;IRb
39	106518	128761	P	30	-2	2.20E-05	IGS	IRb;IRa
40	106550	128793	P	30	-2	2.20E-05	IGS	IRb;IRa

(*Continued*)

Table S3: *Continued*

ID	Repeat I Start	Repeat II Start	Type	Size (bp)	Distance	*E*-Value	Gene	Region
41	128761	128793	F	30	-2	2.20E-05	IGS	IRa;IRa
42	34647	44478	P	30	-3	6.17E-04	*trnS-UGA;trnS-GGA*	LSC;LSC
43	74932	116709	F	30	-3	6.17E-04	*petB;ndhA*	LSC;SSC
